# Is methylmethacrylate toxic during pregnancy and breastfeeding?--- a systematic review

**DOI:** 10.1186/s42836-020-00059-z

**Published:** 2021-02-03

**Authors:** James S. Lin, Janice A. Townsend, Casey Humbyrd, Julie Balch Samora

**Affiliations:** 1 The Ohio State University Department of Orthopaedics, Columbus, OH, USA; 2 Department of Dentistry, Nationwide Children’s Hospital, Division of Pediatric Dentistry, The Ohio State University College of Dentistry, Columbus, OH, USA; 3 Johns Hopkins University Department of Orthopaedics, Baltimore, MD, USA; 4 Nationwide Children’s Hospital Department of Orthopedic Surgery, T2E-A2700, 700 Children’s Drive, Columbus, OH 43205 USA

**Keywords:** Methyl methacrylate, Pregnancy, Maternal fetal health, Bone cement, Toxicity, Orthopaedic surgery, Dentistry

## Abstract

**Introduction:**

Methyl methacrylate (MMA) is commonly used in the fields of dentistry and orthopaedic surgery. However, there remain concerns for the occupational hazards of MMA, particularly during pregnancy and breastfeeding.

**Methods:**

We performed a systematic review of studies on effects that MMA may have in pregnancy in the context of exposure during orthopaedic surgery and dentistry. Review articles, studies lacking statistical data, single case reports and other evidence level V studies were excluded.

**Results:**

Nine studies were included. One basic science study demonstrated an increase in neuronal cell lysis and shrunken cell bodies when neocortical neurons were exposed to MMA monomer. Three animal studies exposed pregnant rodents to MMA via intraperitoneal injection or inhalation. Exposed fetuses in two studies had an increase in gross abnormalities such as hemangiomas, while there was no increase in teratologic effects in the third study. In dental workers exposed to MMA, two retrospective cohort studies did not find a statistically significant increase in birth defects or miscarriage. After exposure to MMA during total joint arthroplasty, two studies found that MMA levels were undetectable in the mothers’ serum or breast milk. One study measuring the airborne levels of MMA during simulated joint arthroplasty found that concentrations never exceeded 1% of the recommended limit set forth by the Occupational Safety and Health Administration (OSHA).

**Conclusions:**

Potential teratologic effects of MMA cannot be excluded by existing evidence. However, the typical MMA exposure levels for dental and orthopaedic personnel appear to be substantially less than currently proposed exposure limits.

**Supplementary Information:**

The online version contains supplementary material available at 10.1186/s42836-020-00059-z.

## Level of evidence

IV

## Background

Methyl methacrylate (MMA) is often used in the healthcare setting as the main constituent in bone cement for fixation of orthopaedic implants as well as restorative and prosthetic resins in dentistry [1, 2]. The MMA monomer is a colorless, pungent liquid metabolized to methacrylic acid, with described toxicities to various tissues [3, 4]. Therefore, the United States Environmental Protection Agency (EPA) and Occupational Safety and Health Administration (OSHA) advise limiting airborne exposure of MMA to 100 ppm (ppm) averaged over 8 h [4, 5]. Similarly, the American Conference of Governmental Industrial Hygienists recommends a limit of 50 ppm over an eight-hour period with 100 ppm as the peak exposure limit [6].

Early animal studies investigating the toxicity of MMA found that high concentrations of MMA vapor were lethal due to respiratory depression [7, 8]. Singh et al [9] intra-peritoneally injected MMA into pregnant rats, and reported dose-related increases in external malformations as well as reduced weight in the fetuses. Pregnant rats exposed to MMA vapor had increased incidence of fetal deaths, hematomas, vertebral abnormalities, and decreased fetal weight and size [10]. In contrast, McLaughlin et al. [3] exposed mice to MMA vapor during pregnancy, and these authors did not detect a difference in abnormal fetuses and fetal deaths in exposed mice compared to unexposed mice.

Since these animal studies [3, 9, 10], there have been a number of investigations examining the levels of exposure of methylmethacrylate in operating room conditions, and there remain concerns for the occupational hazards of MMA, particularly in pregnancy and breastfeeding. The purpose of this study was to ask: What evidence exists to investigate what adverse effects, if any, MMA may have on healthcare providers in pregnancy and breastfeeding?

## Materials and methods

**Fig. 1:**
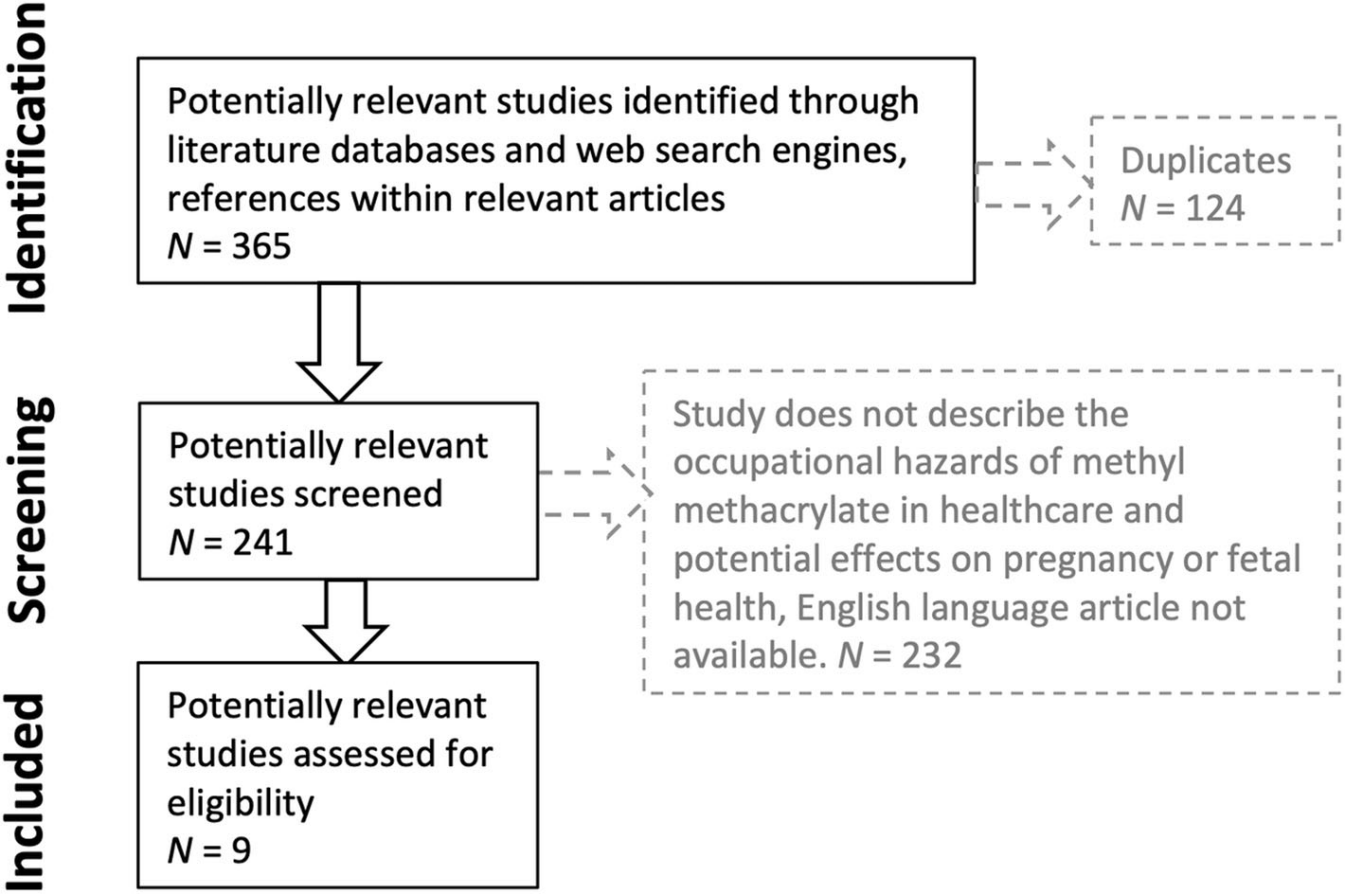
Search strategy according to PRISMA (Preferred Reporting Items for Systematic Reviews and Meta-Analyses) guidelines. Nine studies were identified for inclusion

A systematic review was conducted according to the Preferred Reporting Items for Systematic Reviews and Meta-Analyses (PRISMA) guidelines (Fig. 1) with a search strategy aimed to capture articles reporting outcomes of MMA exposure on pregnancy and breastfeeding. Basic science and animal studies investigating the effects of MMA exposure in pregnancy were also included due to the animal study origins of the initial toxicity profile of MMA and the small number of human studies. Exact search criteria used are reported in Additional files 1. The authors independently confirmed the search on November 27, 2019. The following databases were searched: PubMed, Embase, Web of Science, and The Cochrane Library. References in the relevant studies and results from web search engines were also screened. All abstracts of articles in the initial search were manually reviewed. The full text of all articles with potential for final inclusion was evaluated by the first author. English-language studies on occupational hazards of MMA in the healthcare setting with evidence level IV or higher were included.

## Results

Nine studies were included for final analysis (Table 1). The initial search yielded 365 studies. After removing duplicates, 241 remained. Abstracts were manually screened, and nine articles that mentioned the effects of occupational MMA exposure remained for inclusion. Five [9–12, 14] of the nine studies specifically mentioned dental applications of methyl methacrylate. The other four [3, 13, 15, 16] discussed orthopaedic uses of MMA such as joint arthroplasty, vertebroplasty, and antibiotic beads.

**Table 1:** **Table 1** Studies included in final analysis

Authors	Title	Type of Study	Application of MMA	Results
Singh et al. (1972) [9]	Embryonic-Fetal Toxicity and Teratogenic Effects of a Group of Methacrylate Esters in Rats	Animal study 22 groups of 5 female rats, underwent intraperitoneal injections of methacrylate esters on various days of gestation and at various doses	Denture bases	Fetuses in group exposed to MMA had significant increase in gross abnormalities such as hemangiomas and had significantly lower birth weight. No skeletal malformations were observed with any MMA dose used in this study.
McLaughlin et al. (1978) [3]	Methylmathacrylate: A Study of Teratogenicity and Fetal Toxicity of the Vapor in the Mouse	Animal study 32 pregnant mice 14 control 18 exposed to MMA vapor	Total joint arthroplasty	Slight increase in average weight of fetuses in exposed mothers was noted. No other evidence or fetal toxicity or teratologic effects were found.
Nicholas et al. (1979) [10]	Embryotoxicity and Fetotoxicity from Maternal Inhalation of Methyl Methacrylate Monomer in Rats	Animal study, pregnant rats exposed to MMA vapor 233 fetuses in short exposure 185 fetuses in long exposure 263 fetuses in air control 229 fetuses untreated control	Denture bases	Long exposure to MMA vapor on days 6–15 of gestation had increased incidence of early fetal deaths, hemangiomas, delayed vertebral & sternal ossification and decreased fetal weight and crown-rump length. MMA exposure did not significantly affect incidence of implantations, resorptions, or number of living fetuses per litter.
Chen et al. (1998) [11]	Free Radicals are Involved in Methymethacrylated-Induced Neurotoxicity in Human Primary Neocortical Cell Cultures	Basic science study Exposed embryonic brain issue used for neuro-enriched culture to MMA monomer	Dental implants	Neocortical neurons exposed to MMA had increased lactate dehydrogenase (LDH) levels, irregular shrunken cell bodies, lysis, neurotoxicity.
Dahl et al. (1999) [12]	Dental workplace exposure and effect on fertility	Retrospective cohort study 558 female dental surgeons 450 high school teachers with at least 1 child	Dental surgery, including amalgams and other implants	No difference was found in rates of birth defects. Study does not specifically control for MMA exposure, but addresses dental surgery in general.
Linehan et al. (2006) [13]	Serum and Breast Milk Levels of Methylmethacrylate Following Surgeon Exposure During Arthroplasty	Serum and breast milk analyzed from 2 lactating orthopaedic surgeons after exposure to MMA compared to 2 healthy controls	Total joint arthroplasty	No serum or breast milk sample had detectable MMA levels following inhalational exposure during total joint arthroplasty.
Lindbohm et al. (2007) [14]	Occupational exposure in dentistry and miscarriage	Case controlled retrospective study 222 cases of miscarriage 498 controls (births)	Dental applications	There was a slight but not significant increase in odds for miscarriage for those exposed to MMA in air or polymethylmethacrylate (PMMA) dust.
Homlar et al. (2013) [15]	Serum Levels of Methyl Methacrylate Following Inhalational Exposure to Polymethylmethacrylate Bone Cement	20 healthy volunteers exposed to mixing of PMMA cement in simulated operating room, then serum levels obtained	Joint arthroplasty	MMA was not detected in any of the specimens after exposure during routine mixing of PMMA cement.
Speeckaert et al. (2015) [16]	Airborne Exposure of Methyl Methacrylate During Simulated Total Hip Arthroplasty and Fabrication of Antibiotic Beads	Simulated operating room where the airborne exposure of MMA during total hip arthroplasty and antibiotic beads fabrication was measured	Total hip arthroplasty Antibiotic beads	MMA levels fell to baseline at an average of 11 min post mixing in all total hip arthroplasty simulations. By these authors’ measurements, the MMA exposure was a small fraction of the Occupational Safety and Health Administration limit during an 8 h limit.

Two early animal studies [9, 10] exposing pregnant rats to MMA, via either intraperitoneal or inhalational routes, found significant increases in fetal abnormalities in exposed groups. In 1972, Singh et al studied [9] groups of pregnant female rats. On either day 5, 10, or 15 of gestation, the authors performed intraperitoneal injections of methacrylate esters, including MMA, at a tenth, fifth, or a third of its acute intraperitoneal lethal dose (LD_50_). The authors reported higher gross fetal malformations (such as hemangiomas) with higher doses of MMA. Fetuses from all treated groups of rats had significantly lower weight. No skeletal malformations were observed with any dose of MMA used in this study. A 1979 study by Nicholas and colleagues [10] exposed pregnant rats to a concentration of MMA vapor (110 mg/liter) at one quarter and three quarters of the median lethal time (LT_50_) on day 6 through day 15 of gestation. Long exposure was associated with increased incidence of early fetal deaths, hemangiomas, delayed vertebral and sternal ossification, and decreased fetal weight and crown-rump lengths.

In a similar 1978 animal study, McLaughlin et al. [3] exposed 18 pregnant mice to vapors from evaporating MMA monomer at an average concentration of 1330 ppm for 2 h twice daily from day 6 through day 15 of pregnancy. After sacrifice, the fetuses were examined morphologically, and the authors did not detect any difference in abnormalities, fetal resorptions, or fetal deaths between exposed mothers and controls. In contrast to the study by Nicholas et al. [10], no teratologic changes were noted except for a slight increase in average weight in fetuses of exposed mothers. These authors also investigated the airborne concentration of MMA during cement mixing in the operating room, and found that concentrations never rose above 280 ppm [3].

A basic science study conducted by Chen et al. [11] analyzed cerebral cortex tissue from six human embryos. Cell cultures were performed, and MMA monomer dissolved in glycerol was used to expose the neuron-enriched neocortical cultures for 48 h. The authors found that the MMA monomer exposure induced neuronal damage as measured by lactate dehydrogenase (LDH) levels and also revealed by morphologic analysis (*i.e*. irregular and shrunk cell bodies, fragmented neuritis, and cell lysis).

Since these animal and basic science investigations, studies addressing the occupational exposure to MMA and pregnancy have been mostly clinical in nature. Two retrospective cohort studies [12, 14] found that exposure to dental surgery and dental applications of MMA did not increase rates of pregnancy complications. Dahl et al. [12] performed a retrospective cohort study involving 558 female dental surgeons and 450 high school teachers in Norway who had given birth to at least one child. This study investigated dental surgery in general, surveying the female dental surgeons on their duration of practice. It examined exposure to different chemicals and radiation but did not specifically investigate MMA. These authors concluded that through these questionnaires, occupational exposure did not appear to adversely affect fertility among female dental surgeons compared to high school teachers.

Similarly, a retrospective study by Lindbohm et al. [14] examined 222 cases of miscarriage and 498 controls (births) among female dental workers and a comparison group unexposed to the materials in question. Through analyses of survey data and pregnancy data through hospital records, the authors found a slight but not significant increase in odds for miscarriage for those exposed to MMA in air or polymethylmethacrylate (PMMA) dust. However, the authors acknowledged that dental workers might have simultaneous exposure to a variety of acrylate compounds and also mercury amalgam, so the increased risk could not be ascribed to any one specific compound. They conclude that a possible weak association between exposure to dental restorative materials and miscarriage cannot be excluded by their study.

The other three studies addressed orthopaedic applications of MMA. Linehan and Gioe [13] evaluated two healthy lactating orthopaedic surgeons and two healthy breastfeeding volunteers without exposure to MMA. Between the two surgeons, there were seven total knee arthroplasties and one hybrid total hip arthroplasty performed. Serum and breast milk samples were obtained at various time points (between 11 to 23 min) after start of cement mixing. The authors found that no serum or breast milk sample had detectable MMA at the 0.5 ppm level using headspace gas chromatography, nor did any sample provided by a surgeon have a higher level than the controls. In a similar study, Homlar et al. [15] evaluated twenty healthy volunteers exposed to MMA mixing in a simulated operating room environment without the use of exhaust hoods and an open mixing bowl technique. Two specimens were obtained from each participant during the expected peak inhalational exposure time points (30 s and 3 min after initiation of cement mixing). MMA was undetectable at the 0.5 ppm level.

Finally, Speeckaert et al. [16] measured the airborne exposure of MMA during total hip arthroplasty and antibiotic bead fabrication in an operating room environment. The authors did several experiments. During simulated total hip arthroplasty with vacuum mixing of cement in a hood, the mean peak exposure for the surgeon and scrub technician was 8.4 ppm and 19.5 ppm, respectively, and levels returned to baseline of < 1 ppm at a mean of 8.4 min and 11 min, respectively. This corresponded to 0.15 ppm and 0.49 ppm averaged over 8 h, respectively. When the same experiment was conducted with mixing in a hood, the peak exposure mean for the surgeon and scrub technician was 20 ppm and 17.9 ppm, respectively, with levels returning to baseline at 14.8 min and 7.9 min, respectively. This was approximately 0.34 ppm and 0.27 ppm averaged over 8 h, respectively. During simulated antibiotic bead fabrication with hand mixing outside of a hood, the peak exposure for the surgeon / scrub station was 19.3 ppm with levels returning to baseline at 15.8 min. This exposure corresponded to 0.82 ppm averaged over 8 h. These exposures were all less than 1% of the OSHA maximum exposure limit of 100 ppm for 8 h [17]. While the authors acknowledged that the health effects of this exposure are beyond the limit of their study, they did conclude that the level of MMA exposure during arthroplasty surgery was far below the recommended limit.

## Discussion

The toxicity profile of MMA has largely been developed from animal studies in rodents. Specifically, its potential effects in pregnancy have come from exposing pregnant rats and mice to MMA through intraperitoneal injection or inhalation of vapor at relatively high levels. For instance, McLaughlin and colleagues subjected pregnant mice to concentrations of 1330 ppm for 2 h twice daily for 10 days, whereas the OSHA recommends a maximum exposure of 100 ppm over 8 h [17]. In addition, the studies by Singh et al. [9] and Nicholas et aL. [10] used fractions of lethal doses, with the former using between 10–33% of its acute lethal dose by injection, and the latter using between 25 and 75% of the acute lethal dose by inhalation. Notably, the breathing zone concentration of MMA during total hip arthroplasties never exceeded 280 ppm in the study by McLaughlin et al. [3]. Moreover, in Speeckaert et al’s [16] simulation of cement mixing during total hip arthroplasty and creation of antibiotic beads, the exposure of MMA over 8 h was less than 1% of the OSHA exposure limit. This finding suggests that the exposures of the animals to MMA in the animal studies were significantly higher than what would ever be encountered by surgeons, dentists, or surgical technicians. Therefore, the applicability of these animal studies to workplace conditions must be considered.

In addition, the two clinical studies by Linehan et al. [13] and Homlar et al. [15] did not detect MMA in breast milk or serum of those exposed to the mixing of MMA in the setting of joint arthroplasty. However, Linehan and colleauges [13] acknowledged the limitation that they only had two lactating surgeons as subjects. The authors also contended that they were unable to provide a comprehensive recommendation for pregnant and lactating surgeons regarding exposure to MMA, but the subjects of their study were comfortable with intraoperative MMA exposure during lactation. Homlar et al. [15] attempted to increase the sample size in their study with twenty healthy volunteers exposed to the mixing of MMA in a simulated operating room environment. Although MMA was not detected in any serum sample, the authors did not conclusively assert MMA exposure is safe during pregnancy. However, they do contend that teratologic effects on fetuses are unlikely due to the fact that serum levels of MMA were less than 0.5 ppm after acute exposure.

Despite the data from these aforementioned studies, it has been shown that female orthopaedic surgeons have higher rates of complications in pregnancy, such as increased rates in infertility, preterm labor, preterm delivery, and incidence of congenital anomalies compared to the general United States female population [18, 19]. This finding contrasts with Dahl and colleagues [12], who did not find any clear adverse effects on fertility when female dental surgeons were compared to high school teachers in Norway. In the study by Lindbohm et al. [14], they did not find a strong association between exposure to chemicals used in dentistry and the risk of miscarriage. However, the authors did find a slightly increased but not significant risk with exposure to mercury amalgam, solvents, disinfectants, and some acrylate compounds, including polymethylmethacrylate dust. Therefore, it is important to consider that there are other exposures in the fields of dentistry and orthopaedics that can potentially adversely affect pregnancy besides methyl methacrylate, such as radiation, anesthetic gases, and physical and emotional stress [14, 19, 20].

While fetotoxic effects of MMA may not be well described in humans, other adverse effects of MMA exposure have been noted. Acute toxicity of MMA includes irritation to the skin, eyes, or mucus membranes, which generally occurs at levels around 1000 ppm [4]. At lower levels, MMA has been found to have minor irritating effects on nasal mucosa [21]. For instance, a study by Muttray et al. [21] subjected twenty healthy volunteers to MMA at levels of 50 ppm in an exposure chamber over 4 h and did not note any adverse effects except for minor irritation to the nose. More chronic exposure to MMA among dental workers has also been observed to include dermal irritation such as contact dermatitis [22, 23].

Future work may include investigating the attitudes that professionals in dentistry and orthopaedics have towards pregnancy-related effects of MMA. The study by Linehan et al. [13] contended that Singh’s study [9] demonstrating teratogenicity in rats after intraperitoneal injections of MMA “resulted in the virtual banishment of pregnant women from the operating room during the use of [PMMA]”. However, there were no citations supporting this assertion. A position statement [24] from the Ruth Jackson Orthopaedic Society acknowledged that exposure to radiation and MMA can contribute to challenges faced by female orthopaedic surgeons, but it also cites Linehan’s [13] study which did not detect MMA in serum or breast milk in two breastfeeding surgeons following MMA exposure during joint arthroplasty. Nevertheless, a survey [25] sent to members of the Ruth Jackson Orthopaedic Society revealed a lack of consensus among female orthopaedic surgeons about the risks of MMA during pregnancy and breastfeeding. 90% of respondents claimed that they were familiar with the risks of MMA, while about 40% reported that they would leave the room during MMA use if pregnant [25]. This study also suggests that it is possible that beliefs regarding MMA exposure may deter individuals from pursuing careers where MMA is regularly used [25]. Therefore, it would be worthwhile to investigate if attitudes toward MMA may lead to exclusion of female orthopaedic surgeons in a male-dominated specialty. In contrast, we are not aware of any evidence (anecdotal or otherwise) that advocates for the exclusion of women in dentistry from procedures which require the use of MMA.

### Study limitations

The studies included had considerable variability. Five studies [9–12, 14] investigated MMA with consideration of dental applications, and four studies [3, 13, 15, 16] were performed with discussion of MMA applications in orthopaedic surgery. The large variability between study designs and methods also precluded any type of meta-analysis. The animal studies [3, 9, 10] have questionable applicability to human workplace conditions, as the mice and rats were subjected to doses substantially higher than that experienced by surgeons in the simulated operating room environment. The conclusions of the clinical studies are limited, as the retrospective cohort studies [12, 14] are not able to control for MMA exposure specifically among other agents in the dental workplace. The operating room simulation [16] acknowledged that it was beyond the scope of their study to describe any health effects associated with the level of MMA exposure they found. The two studies investigating serum and breast milk levels of MMA following exposure [13, 15] reported that although MMA levels were undetectable, they cannot make definitive recommendations on its safety during pregnancy.

## Conclusion

While potential teratologic effects of MMA cannot be excluded by existing evidence, the typical MMA exposure levels for dental and orthopaedic personnel appear to be substantially less than currently proposed exposure limits. There is no compelling evidence in the existing literature that suggests that exposure to MMA during routine dental and orthopaedic surgery increases risks for pregnancy-related complications.

## Supplementary Information

**Additional file 1.**

## Data Availability

All data generated or analyzed during this study are included in this published article and its supplementary information files.
