# Supplementary material for: Is methylmethacrylate toxic during pregnancy and breastfeeding?--- a systematic review
Source: Arthroplasty. 2021 Feb 3;3:9. doi: 10.1186/s42836-020-00059-z (PMC8796479; doi:10.1186/s42836-020-00059-z)
Supplement: Supplementary file 1 — Additional file 1. [file 42836_2020_59_MOESM1_ESM.docx]

**Appendix A. Search Terms**

("Polymethyl Methacrylate"[Mesh] OR "polymethacrylic acids"[mesh] OR "methacrylates"[mesh] OR "PMMA-b-PFEMA copolymer" [Supplementary Concept] OR "PMMA"[tw] OR "polymethyl methacrylate"[tw] OR "poly methyl methacrylate"[tw] OR "poly methyl metha crylate"[tw] OR "methyl methacrylate"[tw] OR "bone cement"[tw] OR "bone cements"[tw] OR "palavit"[tw] OR "implast"[tw] OR "kallocryl k"[tw] OR "lucite"[tw] OR "methyl acrylic plastic"[tw] OR "palacos R"[tw] OR "plexiglas"[tw] OR "plexiglass"[tw] OR "superacryl"[tw] OR "acron"[tw] OR "perspex"[tw] OR "polymethylmethacrylate"[tw] OR "polymethylmetacrylate"[tw] OR "methylmethacrylate"[tw] OR "acrylates"[mesh] OR "dental amalgam"[mesh] OR amalgam*[tw] OR "garamycinekogel"[tw] OR "gentamycin acrylate"[tw] OR "septopal"[tw] OR acrylate*[tw] OR "acrylic derivative"[tw] OR "acrylic acid derivative"[tw] OR polymethacrylic*[tw] OR methacrylate*[tw])

AND

("pregnant women"[mesh] OR "fetus"[mesh] OR "abortion, spontaneous"[mesh] OR "pregnant"[tw] OR "pregnancies"[tw] OR "pregnancy"[tw] OR "prenatal"[tw] OR "pre natal"[tw] OR "antenatal"[tw] OR "ante natal"[tw] OR "perinatal"[tw] OR "peri natal"[tw] OR "fetus"[tw] OR "fetuses"[tw] OR "fetal"[tw] OR "miscarriage"[tw] OR "miscarriages"[tw] OR "birth"[tw] OR "births"[tw] OR "lactating"[tw] OR "lactation"[tw] OR "spontaneous abortion"[tw] OR "spontaneous abortions"[tw] OR placenta*[tw])

AND

("Occupational Injuries"[Mesh] OR "Occupational Exposure"[Mesh] OR "Occupational Medicine"[Mesh] OR "dentists, women"[mesh] OR "dentists"[mesh] OR "surgeons"[mesh] OR "oral and maxillofacial surgeons"[mesh] OR maxillofacial*[tw] OR "orthopedic surgeons"[mesh] OR "neurosurgeons"[mesh] OR neurosurgeon*[tw] OR "occupational"[tw] OR "job"[tw] OR "jobs"[tw] OR "employment"[tw] OR "workplace"[tw] OR "work place"[tw] OR surgeon*[tw] OR dentist*[tw] OR provider*[tw] OR personnel*[tw] OR clinician*[tw] OR physician*[tw] OR "dental"[tw])

NOT
("mercury"[ti])
